# Interspecific killing of wolverines by one wolf pack

**DOI:** 10.1002/ece3.10758

**Published:** 2023-12-06

**Authors:** Kiana B. Young, David T. Saalfeld, Colette Brandt, Kyle R. Smith, Timothy J. Spivey, Cory J. Stantorf

**Affiliations:** ^1^ Division of Wildlife Conservation Alaska Department of Fish and Game Anchorage Alaska USA; ^2^ 673 CES/CEIEC Conservation United States Air Force, Joint Base Elmendorf‐Richardson JBER Alaska USA

**Keywords:** Alaska, interference competition, interspecific aggression, interspecific killing, wolf, wolverine

## Abstract

Interactions between different species of predators are not uncommon, yet they are generally understudied in North America. Across their range, gray wolves (*Canis lupus*) and wolverines (*Gulo gulo*) occupy similar habitats and dietary niches. However, due to the elusiveness and relatively low density of these two species, interactions between them are not well documented. Here, we describe three instances of a single wolf pack killing a wolverine in the span of 13 months. None of the wolverines killed by wolves were consumed, suggesting that food was not the primary motivation behind the killings. Alternatively, defense of a food resource, territoriality, interspecific competitive killing, or some combination of those behaviors appear to be the cause of these actions. Documentation of these occurrences improves our understanding of wolf and wolverine ecology, interspecific predator interactions, and potential future changes to this aspect of community ecology.

## Graphical abstract

Here, we document evidence of a single wolf pack in Alaska killing three individual wolverines over the span of 13 months. Because none of the wolverines were consumed, we suggest that food was not the primary motivation for the killings but rather the defense of food or territory or interspecific competitive killing.

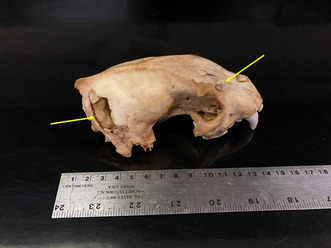

## 1 INTRODUCTION

Predator–prey dynamics have been rigorously studied across many species and ecosystems. These dynamics are known to play crucial roles in shaping the interactions of species within an ecological community (Abrams, 2000; Stevens, 2010). However, the structure of an ecosystem and the interspecific relationships that comprise it go beyond just that of a predator and its prey. Dynamics between predators of different species are less studied, yet they are still relevant to understanding the behavior, distribution, and movement patterns of a species (Abrams, 2000; Kitchen et al., 2011; Tannerfeldt et al., 2002). Competitive interactions between predators can broadly be split into two categories: exploitation interactions and interference interactions (Linnell & Strand, 2008). Exploitation interactions are a type of indirect interaction where a limited resource is shared by two species, and the consumption of that resource by one species precludes the consumption by the other species (Hardin, 1960). Alternatively, interference interactions are direct interactions whereby the dominant species directly prevents the subordinate species from consuming the shared resource, often through aggressive behavior (Case & Gilpin, 1974). Both types of interactions are occurring and likely common in natural populations, but interference interactions in particular are difficult to study in predators due to their elusiveness, wide range, and often lower densities compared to prey species (Smith et al., 2017).

Gray wolves (hereafter “wolves”; *Canis lupus*) are one of the most well‐known apex predators in Alaska. As a social species, they travel and hunt in packs using clearly defined territories, which they regularly guard and defend from other packs (Cassidy et al., 2015; Mech & Boitani, 2003). In Alaska, the main prey items for wolves are ungulates (e.g., moose [*Alces alces*], caribou [*Rangifer tarandus*], Dall's sheep [*Ovis dalli dalli*], mountain goats [*Oreamnos americanus*], and blacktail deer [*Odocoileus hemionus*]), but they have also been known to feed on rodents (e.g., beaver [*Castor canadensis*], muskrat [*Ondatra zibethicus*], etc.), fish (e.g., salmon [*Oncorhynchus* sp.]), birds, snowshoe hares [*Lepus americanus*], and marine mammals (Adams et al., 2010; Roffler et al., 2021; Stanek et al., 2017; Watts & Newsome, 2017). Wolves not only hunt live prey, but they will also scavenge when the opportunity arises (Huggard, 1993). Prior research on wolf interactions with brown bears (*Ursus arctos*) and smaller canid mesocarnivores (i.e., red foxes [*Vulpes vulpes*] and coyotes [*Canis latrans*]) has documented one predator chasing the other off a food resource or away from a den (Lewis & Lafferty, 2014; Terrestrial et al., 2003). One interspecific relationship of interest that is still relatively understudied is that of wolves and wolverines (*Gulo gulo*).

**FIGURE 1:**
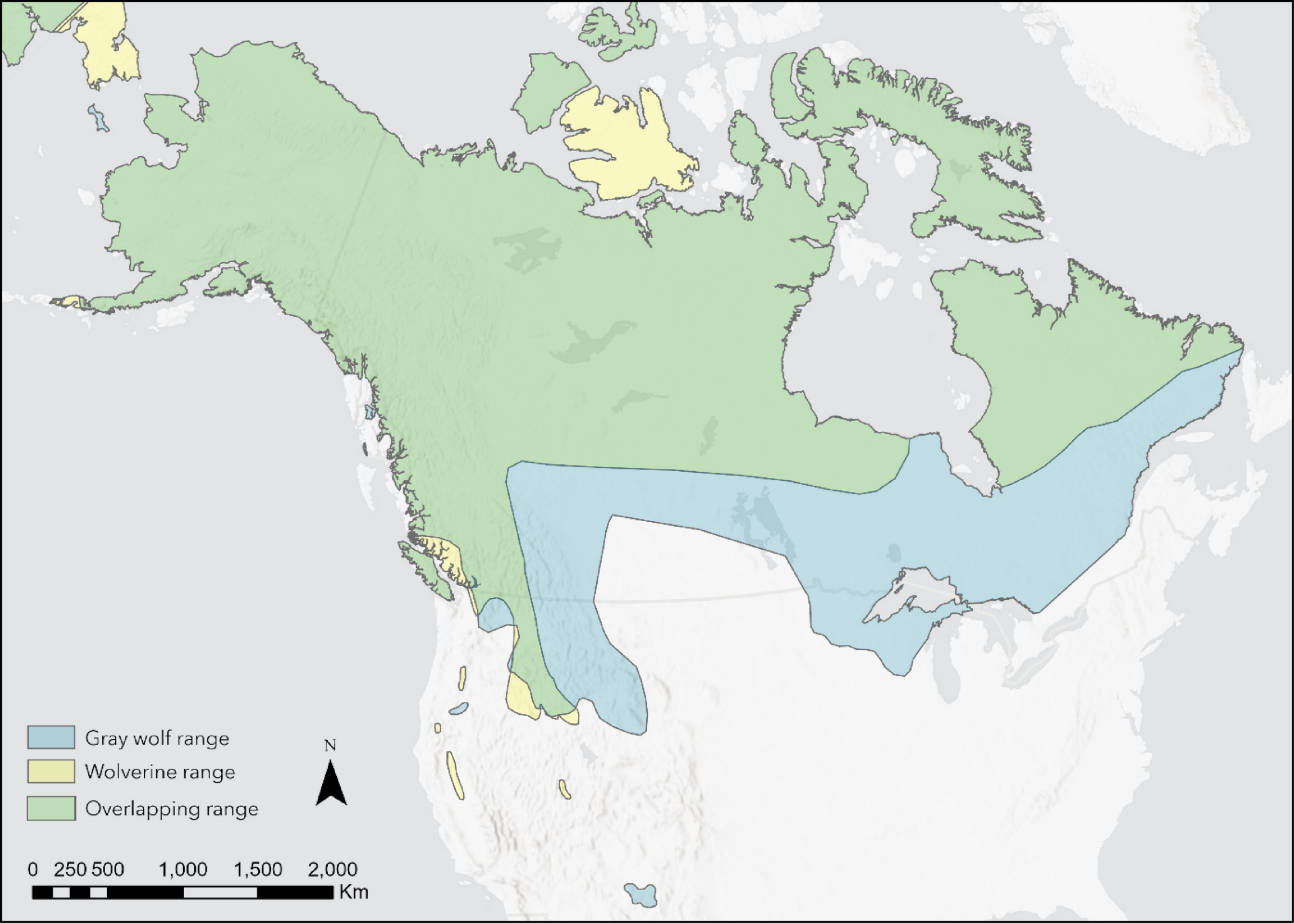
Current geographic range of gray wolves and wolverines in North America based on International Union for Conservation of Nature (IUCN) data (IUCN, 2016, 2018). The green area indicates the area of range overlap between the two species.

Wolves and wolverines encompass much of the same range in North America (Figure 1). Even though wolverines are smaller than wolves (averaging 17 kg vs. 52 kg), they have a similar diet (Dalerum et al., 2009; Lofroth et al., 2007). Wolverines are facultative scavengers, meaning they opportunistically scavenge carrion as a food resource (Mattisson et al., 2016). Wolf‐killed prey provides an opportunity for wolverines to feed, resulting in wolverines shifting their diet when occupying the same area as wolves (van Dijk, Gustavsen, et al., 2008). However, it is thought that wolverines do not necessarily follow wolves to kill sites but rather arrive after the sites have been abandoned to avoid direct interaction with wolves (van Dijk, Andersen, et al., 2008). A dominance hierarchy likely exists between these two species, especially when resources are limited (Chase, 1982). Characteristics including body size, group size, and group composition are involved in the establishment of a hierarchy (Hamilton & Benincasa, 2022; Tibbetts et al., 2022). Wolves are larger than wolverines and form social groups, making them the likely dominant species; however, while interactions between these two species do occur, little is known about their prevalence and outcome.

Here, we describe three instances of a wolf pack killing three separate wolverines. Between 4 January 2022 and 16 April 2023, we had five wolves in one pack collared for a population dynamics study of wolves in Chugach State Park and on Joint Base Elmendorf‐Richardson near Anchorage, Alaska. During this period, four wolverines who had overlapping territories with the wolf pack were also collared, and three of these four wolverines were killed by members of this pack. Because both the wolves and wolverines had global positioning system (GPS) collars and were aerially located quickly after mortality signals were received, we can confidently attribute the cause‐specific mortality of these wolverines to wolves.

## 2 METHODS

### 2.1 Study area

The wolves and wolverines in this study occupied portions of Chugach State Park (~2000 km^2^) and Joint Base Elmendorf‐Richardson (~260 km^2^). This area is generally characterized by a diverse array of mixed forest and alpine tundra vegetation, with the main ecotypes being upland rocky moist mixed forest, upland rocky moist broadleaf forest, alpine rocky dry dwarf scrub, and alpine rocky moist dwarf scrub (U.S. Air Force, 2021). Where vegetation does not occur, snow‐ and ice‐covered peaks up to 2400 meters in elevation represent much of the Chugach Mountains. On both state park and military land, potential prey items for wolves and wolverines include moose, mountain goat, beaver, Dall's sheep, multiple species of salmon, a variety of small mammals, and upland bird species.

### 2.2 Collared animals

Five wolves (three adult males, one subadult male, and one female pup) belonging to a single pack were captured and equipped with GPS collars (Telonics Inc., Mesa, AZ) between March 2021 and March 2022 for a separate study on home range size, pack dynamics, and survival. Collars were programmed to record a location fix every 1.75 h and transmit data every 2 days. These five wolves belonged to a pack that was comprised of 13 individuals in winter 2021/2022 and 11 individuals in winter 2022/2023. Four wolverines in this study area (two adult females, one adult male, and one juvenile male) were captured on 4 January 2022 (Wolverine 2), 12 March 2022 (Wolverine 5), 4 December 2022 (Wolverine 6), and 20 December 2022 (Wolverine 7). These individuals were also fitted with GPS collars that were programmed to record a GPS location every 4 h and send data every 7 days. During the winter months, the collared wolf pack was located from a fixed‐wing aircraft twice a month to assess pack size, and whenever a mortality signal was received from either species, we would attempt to locate and necropsy the carcass as quickly as possible. The likely cause of death was determined using a variety of criteria, including investigating puncture wounds, broken bones, hemorrhaging, signs of disease, and body condition.

### 2.3 Home range estimates

We calculated home range estimates for the wolf pack and the three deceased wolverines to determine the size and amount of overlap between them. GPS points for the five collared members of the wolf pack were trimmed to match the time period of the collared wolverines (4 January 2022–16 April 2023) and then combined to determine the pack home range. All home ranges were calculated using the ‘adeHabitatHR’ package (Calenge, 2006) in R (v4.2.2; R Core Team, 2021) using kernel density estimation with a fixed 95% contour. Home range shapefiles were exported and visualized using ArcGIS Pro (v3.1.1. Redlands, CA: Environmental Systems Research Institute, Inc., 2010).

## 3 RESULTS

### 3.1 Wolverine 2

**FIGURE 2:**
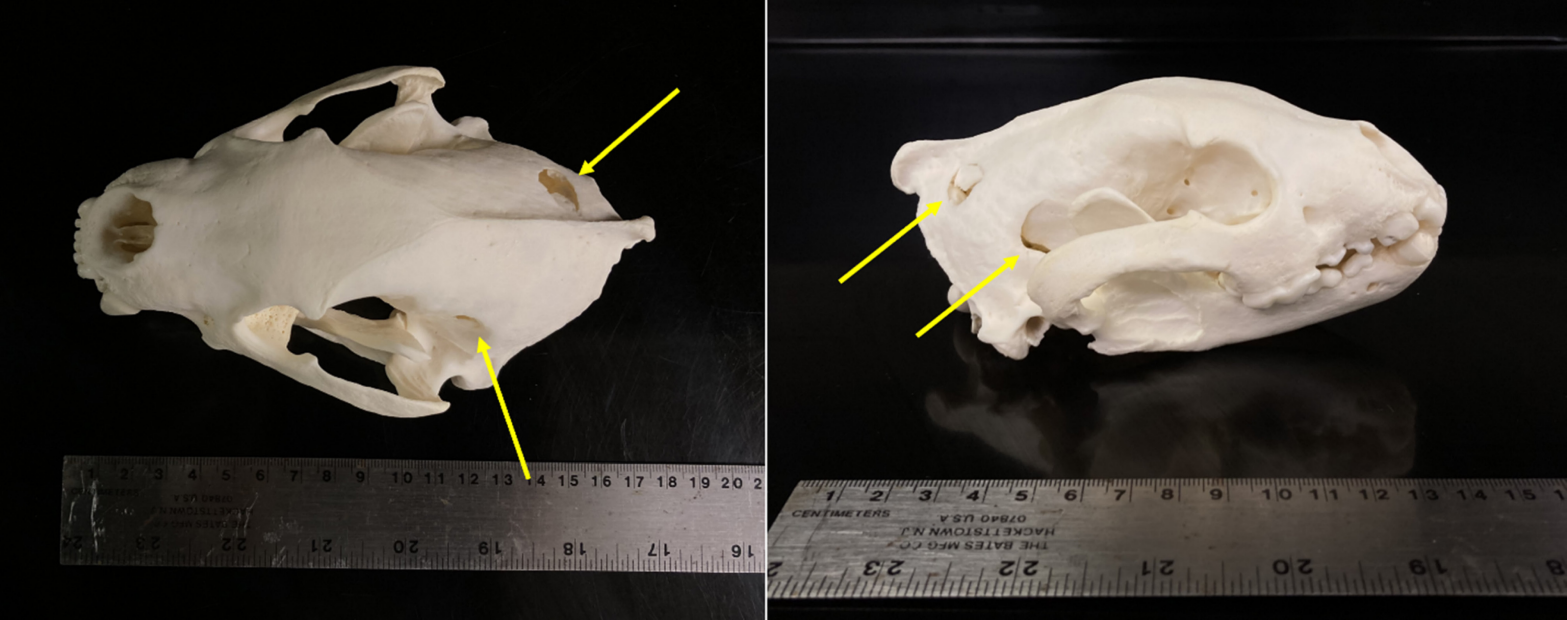
The skull of Wolverine 2, an adult female, that was killed 12 March 2022 in Chugach State Park, Alaska. Yellow arrows indicate skull fractures.

Wolverine 2, an adult female, was found dead in open, snow‐covered habitat during aerial wolf captures on 12 March 2022. While tracking the collared pack of wolves from a helicopter, biologists saw blood and the wolverine carcass in the snow near the wolf pack's location along with wolf tracks surrounding the carcass. The helicopter landed and the carcass was retrieved to collect the collar and perform a necropsy. The collar had not yet sent a mortality signal by the time it was found, indicating that the wolverine was killed early that morning or during the previous night. The necropsy revealed puncture wounds in the hide and skull indicative of wolves, but the wolverine had not been consumed (Figure 2).

### 3.2 Wolverine 7

**FIGURE 3:**
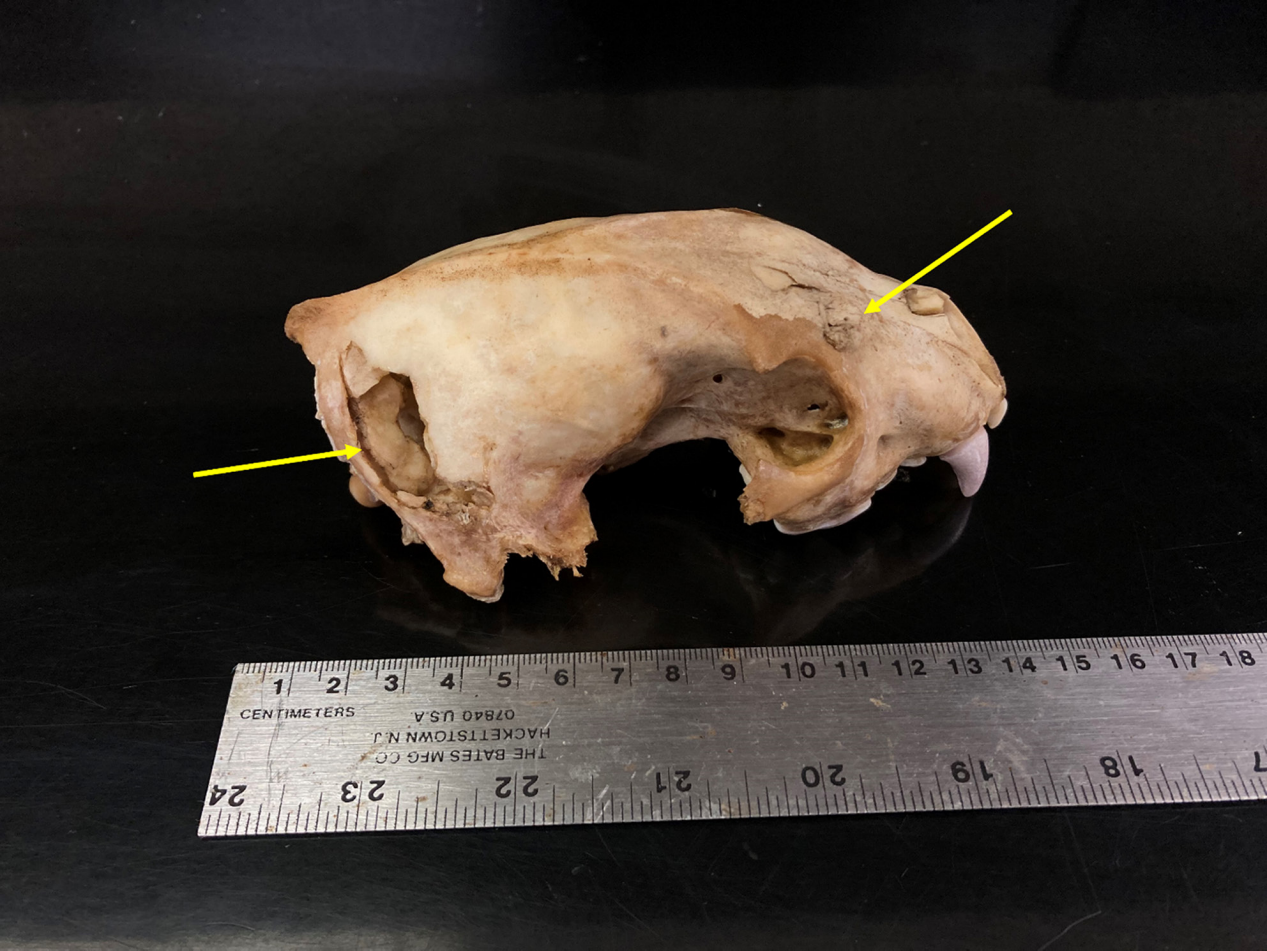
The skull of Wolverine 7, a juvenile male that was killed 30 January 2023 in Chugach State Park, Alaska. Yellow arrows indicate skull fractures.

A mortality signal was received from Wolverine 7, a juvenile male, on 30 January 2023. A fixed‐wing pilot flew the area the same day, and when he located the wolverine carcass, he observed a wolf standing over the carcass with tracks from many wolves in the snow around the carcass. No blood was observed in the snow around the wolverine carcass, though this could be explained by fresh snow, or the wolverine being killed at a different location and brought to this site. Wolverine 7 had also not been consumed at the time of this observation. The carcass was unable to be retrieved for necropsy due to snow conditions immediately following the mortality signal. During one retrieval attempt in May, personnel could not reach the carcass because of an observation of wolves and a family group of four brown bears interacting and acting agitated near the wolverine carcass. The GPS collar and skull of Wolverine 7 were finally retrieved on 1 August 2023, but by this time, all soft tissue was gone and only the skull remained. The skull was found in an open, low‐brush habitat that had been covered by snow at the time of death. Puncture holes consistent with wolf teeth were also found in the skull of this wolverine (Figure 3).

### 3.3 Wolverine 6

We received a mortality signal from Wolverine 6, an adult female, on 16 April 2023. We hiked into the location shortly thereafter but were unable to locate the animal at that time. On 27 April 2023, we located the wolverine carcass by helicopter and landed to retrieve the carcass. Using the GPS location data, we determined that the wolves had been in that area during the time when the mortality signal was first transmitted. Near the carcass, we discovered a wolverine den ~100 meters away at the edge of a forested habitat and a moose kill with an abundance of wolf signs ~300 meters away. There were signs of a struggle around the carcass, including blood, broken branches, and patches of hair on the snow and at the base of nearby trees. The carcass was partially consumed by what we believe to be small scavengers (birds, small mammals, etc.), but we did not see signs that wolves consumed or fed on the carcass.

**FIGURE 4:**
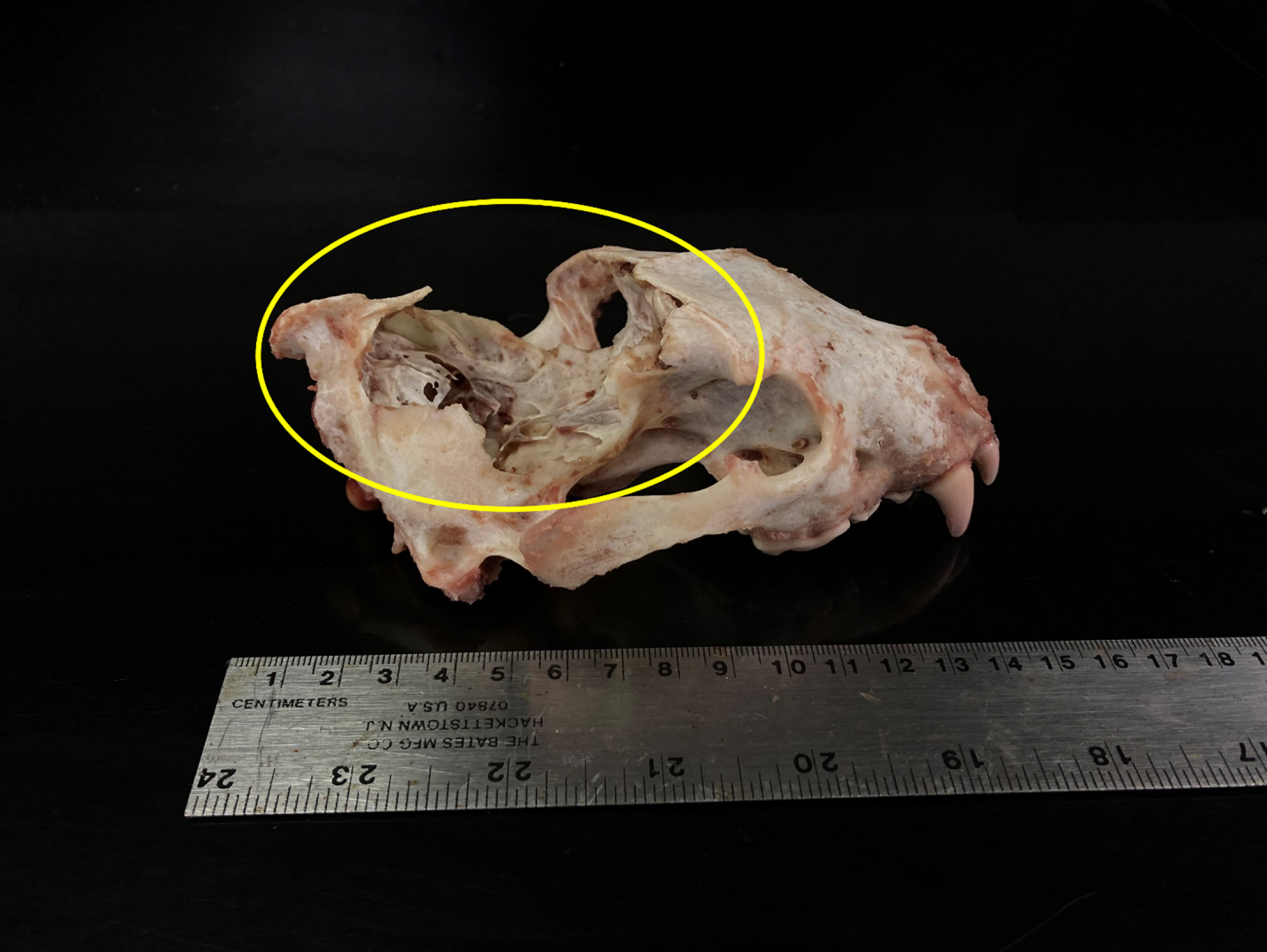
The skull of Wolverine 6, an adult female, that was killed 16 April 2023 in Chugach State Park, Alaska. The yellow circle indicates the fractured section of the skull.

The necropsy of Wolverine 6 revealed puncture wounds consistent with those of a wolf along the neck of the animal. The posterior half of the skull was crushed (Figure 4), and excessive hemorrhaging was present, though the skin was not broken or pierced. This type of damage to the skull could be caused by a fall or a kick from a moose; however, the puncture wounds in the neck and the lack of steep terrain suggest that wolves were responsible for the death and likely swung the wolverine head‐first into a hard object such as a tree.

### 3.4 Home range estimates

**FIGURE 5:**
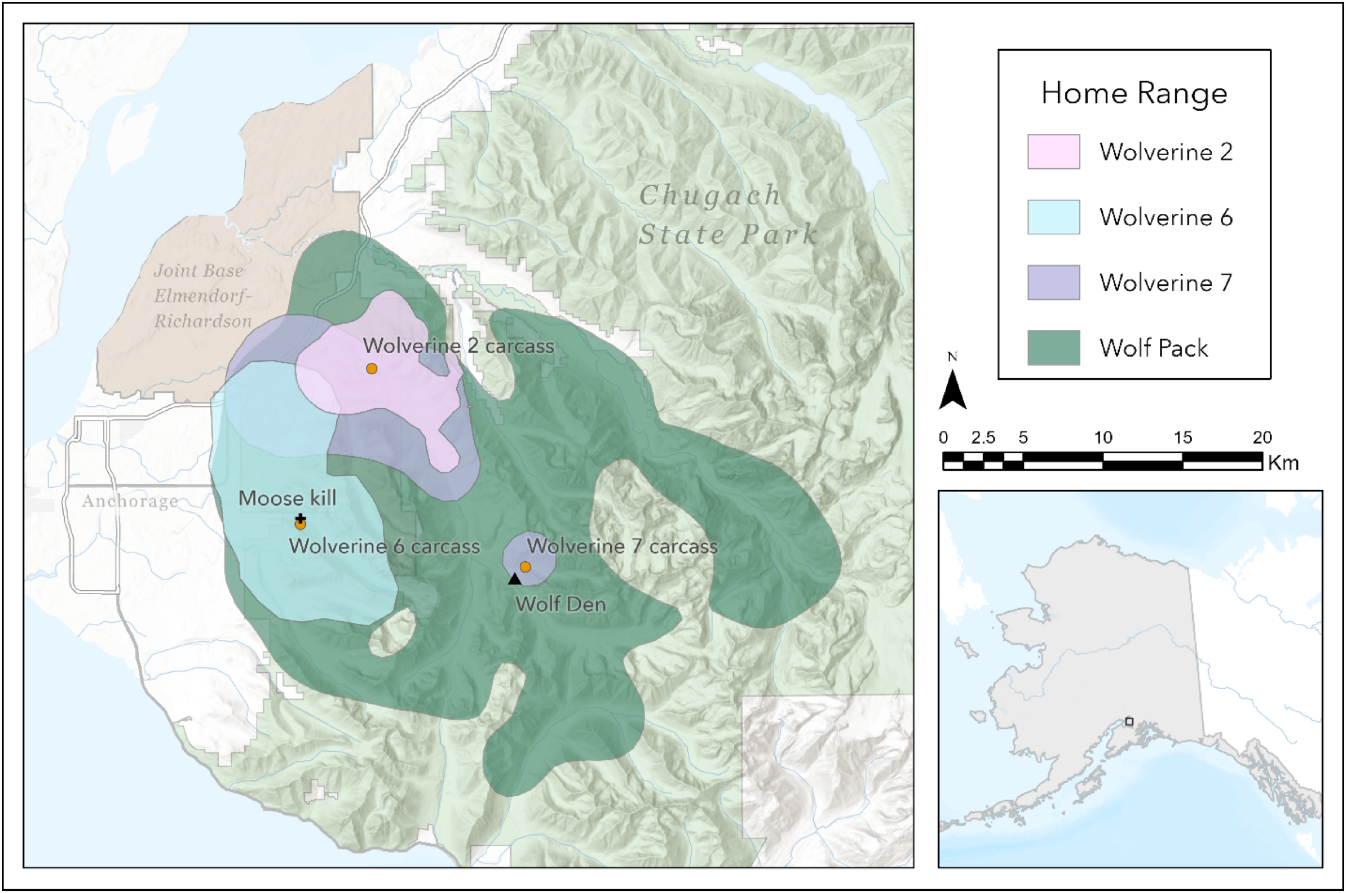
Carcass locations and home range estimates of the wolf pack and three wolverines killed by the wolf pack. All were equipped with GPS collars in Chugach State Park and Joint Base Elmendorf‐Richardson, Alaska, USA, between 4 January 2022 and 16 April 2023.

The wolf pack home range encompassed an area of 679 km^2^. The three wolverine home ranges were smaller (Wolverine 2: 55 km^2^, Wolverine 7: 121 km^2^, and Wolverine 6: 125 km^2^) and fell almost entirely within the boundary of the wolf pack home range (Figure 5). One additional male wolverine had a home range that also fell largely within the wolf pack home range but survived at least until the collar dropped off on 30 June 2023.

## 4 DISCUSSION

There are a variety of possible explanations as to why wolves might kill a wolverine. Wolves are carnivores and rely on meat as their predominant source of food. Consequently, one possibility is that the wolves killed the wolverines for food; however, evidence at each kill site indicated that the wolverine carcasses were mostly unconsumed, so killing for food was likely not the primary motivation. Wolves have been known to eat a variety of different prey items and will shift their diet when ungulates are not available (Roffler et al., 2021; Watts & Newsome, 2017). For example, wolves on a small island in Southeast Alaska have shifted from eating deer to more marine‐based items, notably sea otters (Roffler et al., 2023). While wolverines have been found in wolf scat previously through genetic metabarcoding (Roffler et al., 2021), their low density in this area, in combination with the carcasses that we found unconsumed, makes it unlikely that wolves killed these wolverines for food.

Another possibility for this interspecific aggression is the defense of territory, a food resource, or a den. Wolf packs have distinct territory boundaries that they fiercely defend from other wolves. It is not uncommon for wolves to kill other wolves (Adams et al., 2008; Cubaynes et al., 2014), and while less common, wolves have been documented killing other canids, including coyotes, foxes, and domestic dogs (*Canis lupus familiaris*; Fritts & Paul, 1989; Merkle et al., 2009; Petroelje et al., 2022). Similarly, interactions have been documented between wolves and bears at food resources and dens where wolves both displace bears and are displaced by bears (Gunther & Smith, 2004; Lewis & Lafferty, 2014). Territory defense is less common against smaller non‐canids, such as wolverines, which likely represent less of a safety threat to wolves. However, if the opportunity arises and a wolverine is seen as a threat to the pack or the territory, this explanation could be feasible. One of the wolverines was found near a moose kill (Figure 5), and while a kill site was not observed near the others, that does not mean that one was not present. While the death of Wolverine 7 was prior to the known denning period for wolves, the observed interaction between the wolves and bears in May 2023 did fall within this time period. The den of the wolf pack, which was estimated using the GPS data, was located ~200 m from the observed bear interaction and <1 km from the wolverine carcass (Figure 5). The proximity to the den and timing of this wolf‐bear interaction indicated that this behavior was likely in defense of a den and is an example of the wolf pack showing defensive behavior. One observation to note is that this pack was larger than average, with 11–13 members (Fuller et al., 2003). Previous research has shown that while larger pack sizes do not necessarily result in improved hunting success (MacNulty et al., 2012), larger wolf packs are more successful at capturing difficult and dangerous prey (MacNulty et al., 2014). Additionally, wolf inter‐pack aggression is positively correlated with pack size and the number of prime‐age and male individuals (Cassidy et al., 2015). Although it is not currently known if larger packs exhibit higher interspecific aggression, this pattern would not be surprising given the increased intraspecific aggression and predation on difficult prey that have been previously documented.

The one collared wolverine that was not killed during the study period was a large male. It is possible that, due to its size relative to the other three wolverines, this individual was able to escape or avoid interaction with the wolves while the other three could not. It is also possible that because this wolverine had a larger home range size (744 km^2^) than the other three, it was less likely to come into close proximity with the wolf pack.

Wolves have also been known to exhibit interspecific competitive killing, which is the killing of another species at the same trophic level for the purpose of removing competition from the landscape (Berger & Gese, 2007; Newsome & Ripple, 2015; Polis & Holt, 1992). This form of interference competition differs from intraguild predation because the victim of the lethal interaction is not consumed (Lourenço et al., 2014). Because wolverines feed on many of the same species and often scavenge wolf kills, the removal of the wolverines by the wolves allows for more food availability for the wolves, potentially resulting in higher survival and fitness for the pack if food is limited (Kimbrell et al., 2007). If a wolverine was close to a wolf kill as was observed with one of the collared wolverines, the wolves may have seen the wolverine feeding on the carcass or traveling through the area to or from a den and had the opportunity to attack and kill that individual.

While we cannot say with certainty the reasoning behind this wolf pack killing multiple wolverines, it is likely a combination of territory and food resource defense and interference competition. Prior evidence of wolves killing wolverines has been documented in Alaska on a few occasions. On one account, a group of wolves was observed digging in the snow with blood around a suspected wolverine den site (White et al., 2002). The wolves were not observed killing or consuming any wolverines, but the amount of blood suggested multiple kits were killed (White et al., 2002). Another account describes an observation of two wolves circling a dead female wolverine, which was later necropsied and found to have puncture wounds that likely matched wolf bite marks (White et al., 2002). In this case, the wolverine was not consumed, and based on the behavior and timing of this event, it is believed the wolves were likely defending a den site (White et al., 2002). These prior incidents represent only two isolated events in the literature, and, to our knowledge, the killing of multiple wolverines by a single wolf pack that we describe here has not been documented before, but as the landscape changes, the frequency of these interactions has the potential to increase.

Future changes to the landscape could continue to alter the dynamics between these two species. The impacts on habitat caused by climate change and the expansion of human development could both change the prevalence of these interactions. For example, the lower levels of snowfall that are predicted under climate change models (Bigalke & Walsh, 2022) could decrease the hunting success rate of ungulates by wolves. In the winter, wolves have a higher success rate hunting moose when the snow is deeper and moose are in poorer condition (Huggard, 1993; Post et al., 1999; Sand et al., 2012). Prey availability is an important factor in wolf territoriality, and less available or accessible prey likely leads to increased competition between wolf packs and other species with a shared dietary niche (Martins et al., 2020; Tallian et al., 2022). Wolverines also rely heavily on snowpack for denning and resting (Glass, Breed, Liston, et al., 2021); changes in snow conditions could cause a higher risk of interspecific interactions where deep snow is not available to provide protection or there is a change in animal movement that results in a concentration of snow‐reliant species (Glass, Breed, Robards, et al., 2021). In addition to changes in the climate, increased human development could affect these dynamics by encroaching into valuable habitat and decreasing the prey availability and movement corridors for both wolves and wolverines (Muhly et al., 2019). Changes to the natural landscape are inevitable, but as we address here, gaining an understanding of the current dynamics between these two species can be valuable in the conservation and management of the species.

These findings improve our understanding of both wolf and wolverine ecology in Alaska. Furthermore, because of the low population density of wolverines in this area (4.9 animals/1000 km^2^; Golden et al., 2017), the loss of three individuals in a single year could have a substantial impact on the population. Additionally, three wolf‐killed wolverines within a short period of time within this wolf pack's territory suggests that this wolf pack may have developed a specialized skill in killing wolverines or that instances of wolves killing wolverines could be more prevalent than previously expected.

## AUTHOR CONTRIBUTIONS

**Kiana B. Young:** Conceptualization (lead); formal analysis (lead); investigation (lead); methodology (lead); visualization (lead); writing – original draft (lead); writing – review and editing (equal). **David T. Saalfeld:** Conceptualization (equal); funding acquisition (equal); investigation (equal); methodology (equal); project administration (lead); resources (lead); supervision (lead); writing – review and editing (equal). **Colette Brandt:** Conceptualization (equal); funding acquisition (equal); investigation (equal); project administration (equal); resources (equal); writing – review and editing (equal). **Kyle R. Smith:** Conceptualization (equal); investigation (equal); writing – review and editing (equal). **Timothy J. Spivey:** Conceptualization (equal); investigation (equal); writing – review and editing (equal). **Cory J. Stantorf:** Conceptualization (equal); investigation (equal); writing – review and editing (equal).

## CONFLICT OF INTEREST STATEMENT

The authors declare no competing interests.

## Data Availability

GPS location data availability is restricted under Alaska Statue 16.05.815(d). Data for this project can be made available upon reasonable request from the Alaska Department of Fish and Game and is subject to a data sharing agreement.
